# Stair Climbing Ability and Identification of the Nine Stairs Ascent and Descent Test Cut-Off Points in Hip Osteoarthritis Patients: A Retrospective Study

**DOI:** 10.7759/cureus.41095

**Published:** 2023-06-28

**Authors:** Sophia Stasi, Michail Sarantis, George Papathanasiou, George Evaggelou-Sossidis, Magda Stamou, Dimitrios Tzefronis, George Macheras

**Affiliations:** 1 Laboratory of Neuromuscular and Cardiovascular Study of Motion (LANECASM) Physiotherapy Department, University of West Attica (UNIWA), Athens, GRC; 2 7th Orthopaedic Department, Henry Dunant Hospital, Athens, GRC; 3 Department of Minimal Invasive Orthopaedic Surgery, Athens Medical Center, Athens, GRC

**Keywords:** threshold values, roc analysis, reference values, stair climbing, hip osteoarthritis

## Abstract

Objectives

As the prevalence and incidence of hip osteoarthritis (hip OA) continue to rise, measuring the impact of hip OA severity on a patient's functionality is essential. Stair walking is a particularly relevant task to assess hip OA patients, as difficulty with stair ascent is one of the driving factors in deciding to undergo a total hip arthroplasty. Although stairs tests often arise in post-arthroplasty measures, there is a lack of reported stairs performance time in hip OA patients. Therefore, this retrospective study aimed to report the stair performance time of hip OA patients categorized by disease severity and determine cut-off points that differentiate between severity grades.

Materials and methods

The patient selection was based on the review of de-identified data from our research laboratory database. 254 hip OA patients (aged ≥ 50 years) were divided according to the Kellgren-Lawrence classification system into three groups: Grade 2 (n=68), Grade 3 (n=109), and Grade 4 (n= 68). The stair-walking ability was evaluated using the 9S-A/D test. The time taken to ascend and descend the stairs was measured separately, and the total time (9S-A/D) was also recorded.

The one-way ANOVA model, Welch test, Games-Howell posthoc test, Chi-Square tests, and Two-Way ANOVA model were used for the statistical analysis of the data. The cut-off points were obtained by receiver operating curve (ROC) analysis. The statistical significance was set at p<0.05.

Results

Homogeneity was found between the three groups regarding demographic and clinical characteristics, except age and gender (p<0.001). The comparison of the variables (9S-ascent, 9S-descent, and 9S-A/D) between groups, adjusted for gender and age, showed significant differences: Grade 2 individuals had shorter performance times compared to those in Grade 3 and Grade 4 (p<0.005). Simultaneously, patients with Grade 3 hip OA have a shorter performance time than those with Grade 4 hip OA (p<0.005). Regarding ROC analysis of Grade 2 versus Grade 3: The AUCs of 9S-ascend, 9S-descent, and 9S-A/D were 0.742 (95%CI 0.67-0.81), 0.734 (95%CI 0.66-0.81), and 0.745 (95%CI 0.54-0.90), respectively (all p values <0.005). The cut-off points of 9S-ascend, 9S-descent, and 9S-A/D were 8.7 s (sensitivity 56%, specificity 88%), 7.1 s (sensitivity 58%, specificity 80%), and 16.25 s (sensitivity 54%, specificity 90%), respectively. Concerning ROC analysis of Grade 3 versus Grade 4: The AUCs of 9S-ascend, 9S-descent, and 9S-A/D were 0.702 (95%CI 0.62-0.78), 0.711 (95%CI 0.63-0.79), and 0.715 (95%CI 0.64-0.80), respectively (all p values <0.005). The cut-off points of 9S-ascend, 9S-descent, and 9S-A/D were 11.5 s (sensitivity 66%, specificity 65%), 8.3 s (sensitivity 71%, specificity 62%), and 19.05 s (sensitivity 71%, specificity 61%), respectively.

Conclusions

The study provides evidence that the progression of hip OA affected stair walking; the performance time of 9S-ascent, 9S-ascent, and 9S-A/D tests was significantly longer as the severity of hip OA worsened. ROC analysis results show tests' ability to distinguish the cut-off point between different hip OA grades. However, further research is required for the reporting and classification of stair performance time values in hip OA patients and to further investigate the ability of 9S-ascent, 9S-descent, and 9S-A/D tests to predict the grade of hip OA.

## Introduction

Hip osteoarthritis (OA) is among the most prevalent and disabling forms of joint disorder affecting older adults [1]. Worldwide, it was reported that the age-standardized incidence rate of hip osteoarthritis was 18.70 per 100,000 persons, 18.35 per 100,000 males, and 19.03 per 100,000 females [1], while in Europe, 6.1% of people aged 65-80 years had clinical OA of the hip [2]. In the Greek population, hip OA has a prevalence of 0.9/1000: 1.5/1000 in women and 0.3/1000 in men [3]. As the prevalence and incidence of the disease continue to rise, it is essential to understand and accurately measure the impact of the severity of OA on functionality. This is an important component of any orthopaedic or clinical practice [4].

In clinical practice, both patient-reported outcomes (PROs) and physical performance measures (PPMs) were used because they offered complementary information, assessing different aspects of a patient's functional status [5]. Stair walking is increasingly being used as a PPM directly related to function. Several variations of this PPM are used concerning the number of stairs, the performance pace, and the use or not of handrails [6]. Of those, the "9 stairs ascent and descent" (9S-A/D) test was suggested for hip and knee OA patients [7]. Stair negotiation is known as one of the most demanding tasks among the activities of daily living (ADLs) [8]; it has been related to independence and community participation [9], and it is often used as part of therapeutic exercise programs [6]. Stair walking is a particularly relevant task to assess people with hip OA, as difficulty with stair ascent is one of the driving factors for patients deciding to undergo a total hip arthroplasty [10].

Although stairs tests often arise in measures of physical performance post-arthroplasty, there is a lack of reported stairs performance time in hip OA patients. In a recent systematic review of "timed stair tests", in which 88 studies were included [6], only two studies examined the stair ascent/descent test in hip OA patients [11,12]. The reporting and classification of stair performance time values in hip OA patients may contribute to monitoring the impact of the disease's progression on functionality, setting the goals of physiotherapy intervention, and offering the possibility of pre- and post-arthroplasty stair walking performance comparisons.

Therefore, the purpose of our study was to report the performance time values of stair walking in hip OA patients according to the severity of hip OA.

## Materials and methods

Trial design

This study was a retrospective study based on the review of de-identified patients' data collected through the Laboratory of Neuromuscular and Cardiovascular Study of Motion (LANECASM) database. The study conformed to the "Strengthening the Reporting of Observational Studies in Epidemiology" (STROBE) statement for reporting observational studies [13].

Patient selection

The LANECASM data from 2017 to 2018 was used to select and include patients aged 50 and older. The main inclusion criterion was the existence of unilateral and primary hip OA (grades 2, 3, and 4) according to the radiographic Kellgren-Lawrence classification system [14]. Participants were excluded if they had undergone any surgical intervention to the affected hip; had disorders of the other hip, knee, or ankle joint pain; had any neurological condition that may affect lower extremity function; or were taking medication that adversely affected their postural or dynamic balance [15,16]. The collected data included demographics and clinical characteristics of the patients.

Outcome measures and procedures

The "9 stairs ascent and descent" (9S-A/D) test evaluated the stair-walking ability of our participants. The 9S-A/D test was developed for hip and knee OA patients [7]. This test measures the time (in seconds) needed to ascend and descend a flight of nine stairs with a step height of 20 cm. A shorter performance time represents better functionality, and it can detect pre- and post-intervention clinical changes in orthopaedic patients, a difference in performance time of around two seconds [7].

The correct procedures for the 9S-A/D test were carefully explained before a single pilot test. The test was performed only once to minimize habituation bias and avoid affecting the participant's performance. The same researcher recorded all the test performance times using a timer with an accuracy of 1/100 s. Participants were allowed to use the stair's handrail if necessary, but no verbal encouragement or personal assistance was given. Participants were asked to perform the 9S-A/D test in their usual manner, at a safe and comfortable pace. Stair ascent timing was started once the participant lifted their leading foot from the base of the stairs. The timing was stopped when the participant placed both feet on the ninth step. After a 20-second rest period, participants were requested to descend the stairs. The stair descent timing started when the leading foot began lifting from the ninth step and stopped when both feet were placed on the floor. The times taken to ascend and descend the stairs were measured separately, and the total time (ascend/descend) was also recorded.

Statistical analysis

Data were expressed for continuous variables as mean ± standard deviation (SD) and for categorical variables as frequencies (percentages). The Kolmogorov-Smirnov test examined the normal distribution of the parameters.

The comparison of quantitative and qualitative demographic and clinical variables between the grades of the Kellgren-Lawrence classification system (2-3-4) was performed using the one-way ANOVA model, together with the Bonferroni test for pairwise comparisons, the Welch test in cases of violation of homogeneity of variance between groups, and the Chi-Square test, respectively.

The comparison of the 9S-ascent, 9S-descent, and 9S-A/D variables between Kellgren-Lawrence grades' groups was performed using the one-way ANOVA model and Bonferroni test for pairwise comparisons, or the Welch test in cases of violation of homogeneity of variance between groups, together with the Games-Howell post-hoc test for pairwise comparisons. 

Two-Way ANOVA model using as factors the grades of the Kellgren-Lawrence classification system (2-3-4), the gender (male-female), and as covariates the age for the comparison of 9S-ascent, 9S-descent, and 9S-A/D variables between Kellgren-Lawrence grades adjusted for gender and age.

The receiver operating curve analysis (ROC analysis) was used to examine the prognostic ability of 9S-ascent, 9S-descent, and 9S-A/D variables to discriminate patients with different Kellgren-Lawrence grades (2 versus 3 and 3 versus 4) of hip OA, calculating the area under the curve (AUC). The AUCs with their standard error and 95% CI were calculated using the maximum likelihood estimation method, calculating the sensitivity and specificity of the different cut-off points of the 9S-ascent, 9S-descent, and 9S-A/D tests to quantify the ability to discriminate patients between two grades of Kellgren-Lawrence (2 versus 3, and 3 versus 4). The AUC values were interpreted as follows: AUC ≤0.5 "no discrimination", 0.6≥AUC>0.5 "poor discrimination", 0.7≥AUC>0.6 "acceptable discrimination", 0.8≥AUC>0.7 "excellent discrimination", and AUC>0.9 "outstanding discrimination" [17]. The selection criteria for the cut-off points were the maximization of the sum of sensitivity and specificity for each classifying variable. This, in turn, maximizes the Youndex Index, which results in the maximum difference between the test's true positive and false positive rates [18].

All tests are two-sided, and statistical significance was set at p < 0.05. All analyses were carried out using IBM Corp. Released 2012. IBM SPSS Statistics for Windows, Version 21.0. Armonk, NY: IBM Corp.

## Results

Descriptive and clinical data

Two hundred and forty-five hip OA patients were included in the present study. According to the Kellgren-Lawrence classification system, 68 patients were classified as Grade 2 hip ΟΑ, 109 patients as Grade 3, and 68 patients as Grade 4 (Table 1).

**Table 1:** Descriptive and clinical characteristics of the participants The continuous variables were expressed as mean ±standard deviation (SD) and the categorical variables as frequencies (percentages). ^1^ One-way Anova, ^2^ Welch test, ^3^ Chi-square test ​​​​​​​* p<0.005 vs. Grade 2

Characteristics	Grade 2 (n=68)	Grade 3 (n=109)	Grade 4 (n=68)	p-value
Age (years) ^1^	62.13±7.03	66.40±8.51^*^	66.78±8.38^*^	0.001
Gender [n (%)] ^3^				
Men/Women	31 (45.6)/37 (54.4)	24 (22.0)/85 (78.0)^ *^	13 (19.1)/55 (80.9)^ *^	0.001
Height (m) ^1^	1.67±0.08	1.65±0.09	1.64±0.08	0.102
Weight (Kg)^ 1^	76.94±11.28	75.73±13.47	77.53±15.65	0.669
BMI (Kg/m^2^)^ 2^	27.49±3.32	27.70±4.24	28.67±4.42	0.182
Dominant lower limb [n (%)] ^3^				
Right/Left	61 (89.7)/7 (10.3)	91 (83.5)/18 (16.5)	62 (91.2)/6 (8.8)	0.257
Affected hip joint [n (%)] ^3^				
Right/Left	33 (48.5)/35 (51.5)	58 (53.2)/51 (46.8)	39 (57.4)/29 (42.6)	0.587

It was observed that the compared groups (Grade 2, Grade 3, and Grade 4) are homogeneous concerning demographic and clinical characteristics, except for age (p=0.001) and gender (p=0.001) (Table 1). Regarding age, participants with Grade 2 hip OA were statistically significantly younger than those with Grade 3 (p<0.005) and Grade 4 (p<0.005), respectively. Regarding gender, the Grade 2 hip OA subgroup included a statistically significantly smaller number of women than those with Grade 3 (p<0.005) and Grade 4 (p<0.005), respectively (Table 1).

Outcome measures

It was observed that there is no statistically significant interaction between the factors Kellgren-Lawrence grade and gender for all variables, 9S-ascend (p=0.224), 9S-descend (p=0.100), and 9S-A/D (p=0.148) (Table 2).

**Table 2:** Comparison of variables between groups, adjusted for gender and age Two-way ANOVA model using as factors the Kellgren-Lawrence grade and the gender and as covariates the age ^1^ Variables are presented as the mean ±standard deviation (SD) * p<0.05 vs. Grade 2, ** p<0.005 vs. Grade 2, †† p<0.005 vs. Grade 3

Outcome measures	Grade 2 (n=68)	Grade 3 (n=109)	Grade 4 (n=68)	p-value	Interaction between Kellgren-Lawrence grade and gender
9 stairs ascend (s) ^1^	7.42±0.51	9.37±0.47^**^	12.97±0.63^** ^^††^	<0.005	p=0.224
9 stairs descend (s)^ 1^	6.17±0.50	7.63±0.47	11.92±0.62^** ^^††^	<0.005	p=0.100
9 stairs ascend /descend (s) ^1^	13.39±0.96	16.84±0.89^*^	24.89±1.19^** ^^††^	<0.005	p=0.148

There is a difference between the compared groups in relation to the variables 9S-ascend (p<0.005), 9S-descend (p<0.005), and 9S-A/D (p<0.005). Regarding the 9S-ascent test, Grade 2 individuals statistically have a significantly shorter performance time compared to those in Grade 3 (p<0.005) and Grade 4 (p<0.005). Simultaneously, patients with Grade 3 have a shorter performance time compared to those with Grade 4 (p<0.005) (Table 2). In the 9S-descent test, people with Grade 4 statistically have a significantly longer time than those with Grade 2 (p<0.005) and Grade 3 (p<0.005), respectively (Table 2). Concerning the 9S-A/D test, individuals with Grade 2 statistically have a significantly shorter time compared to those with Grade 3 (p<0.05) and Grade 4 (p<0.005), and those with Grade 3 also have a shorter time in relation to those with Grade 4 (p<0.005) (Table 2).

ROC analysis

Grade 2 Versus Grade 3

**Figure 1:**
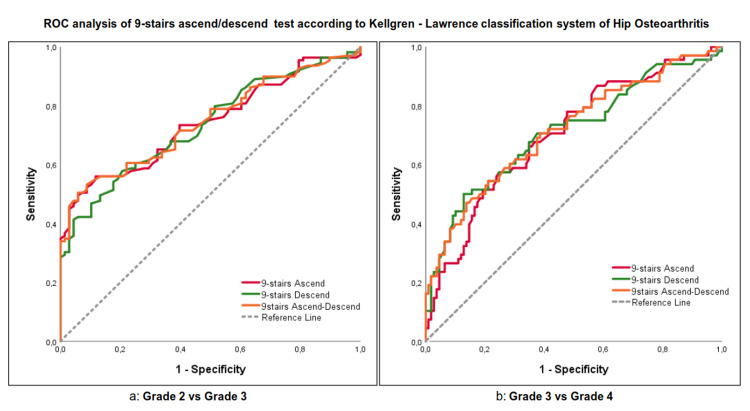
The ROC analysis shows the ability (sensitivity and specificity) of the 9S-ascent, 9S-descent, and 9S-A/D tests to discriminate the severity of hip osteoarthritis according to the Kellgren-Lawrence classification system. a. Grade 2 versus Grade 3, b. Grade 3 versus Grade 4.

The AUC value of the 9S-ascend showed that if the performance time of the test is over the cut-off point of 8.7 seconds (s), there is a 56% chance that the patient has Grade 3 hip OA, while if the time is shorter than 8.7 s, there is an 88% probability of having Grade 2 (Table 3, Figure 1). The AUC of the 9S-descent revealed that when the performance time is higher than the cut-off point of 7.1 s, there is a 58% chance of having Grade 3, while if it is lesser, there is an 80% chance of having Grade 2 (Table 3, Figure 1). As for the 9S-A/D, a performance time higher than the cut-off point of 16.25 s corresponds to a 54% chance that the patient has Grade 3 hip OA, while if the performance is shorter, there is a 90% chance of having Grade 2 (Table 3, Figure 1).

**Table 3:** ROC analysis to distinguish the grade of hip osteoarthritis ^1^ longer performance’s time indicates Grade 3 ​​​​​​​^2^ longer performance’s time indicates Grade 4

Grade 2 versus Grade 3							
Variables	AUC	SE	p-value	Cut-off point (s)	Sensitivity	Specificity	95%CI of AUC
9 stairs ascend	0.742	0.036	<0.005	8.7^1^	56%	88%	0.67 - 0.81
9 stairs descend	0.734	0.037	<0.005	7.1^1^	58%	80%	0.66 - 0.81
9 stairs ascend/descend	0.745	0.036	<0.005	16.25^1^	54%	90%	0.68 - 0.82
Grade 3 versus Grade 4							
Variables	AUC	SE	p-value	Cut-off point (s)	Sensitivity	Specificity	95%CI of AUC
9 stairs ascend	0.702	0.040	<0.005	11.15^ 2^	66%	65%	0.62 - 0.78
9 stairs descend	0.711	0.041	<0.005	8.3^ 2^	71%	62%	0.63 - 0.79
9 stairs ascend/descend	0.715	0.041	<0.005	19.05 ^2^	71%	61%	0.64 - 0.80

Grade 3 Versus Grade 4

The 9S-ascend's AUC showed a cut-off point of 11.5 s, which means that if the performance time is higher than 11.5 s, there is a 66% probability that the patient has hip OA Grade 4; a lesser performance time corresponds to a 65% chance of having Grade 3 (Table 3, Figure 1). The cut-off point of the 9S-descend test is 8.3 s; when the performance time is higher, there is a 71% probability of having Grade 4, while if it is lower, there is a 62% probability of having Grade 3 (Table 3, Figure 1). Finally, the cut-off point of the 9S-A/D AUC is 19.05s, with a 71% probability of having Grade 4 when the performance time is higher, while if it is less, there is a 61% probability of having Grade 3 (Table 3, Figure 1).

In descending order of clinical significance, the 9S-A/D (Σ=1.44), the 9S-descend test (Σ=1.38), and the 9S-ascend test (Σ=1.36) are able to separate Grade 2 from Grade 3 hip OA. In the case of Grade 3 and Grade 4 hip OA, the 9S-descend (Σ=1.33) appears to be better, followed by the 9S-A/D (Σ=1.32) and the 9S-ascend test (Σ=1.31).

## Discussion

The purpose of the present study was to report the performance time values of the 9S-ascent, 9S-descent, and 9S-A/D tests in hip OA patients and to provide cut-off values for these tests according to the severity of hip OA. The results confirm the study's hypothesis, namely that the severity of hip OA affected stair negotiation. This is the first study to provide categorized performance time values for hip OA patients according to the Kellgren-Lawrence grades and showed that in all tests, the performance time was significantly longer as the grade of hip OA was more severe.

Risk factors for developing hip OA are age greater than 60 years, sex (female), excess body weight, genetic factors, and occupations involving heavy manual work and high-impact sports activities [19-21]. The mean age of all study groups was greater than 62 years; 72.3% of our sample was female, and according to BMI results, our participants were in the overweight range; participants of the Grade 2 group were classified as pre-obese-I, while participants of Grade 3 and 4 groups were classified as pre-obese-II [22]. According to these descriptive characteristics, our participants were an adequately representative sample of hip OA patients. Our sample's characteristics also support previous studies that reported that symptoms and disability increase in prevalence with increasing age [21,23] since the study's participants with Grade 2 hip OA were statistically significantly younger than those with Grade 3 (p<0.005) and Grade 4 (p<0.005), respectively. 

Our results showed that the progression of the hip OA affected the performance of the 9S-ascent, 9S-ascent, and 9S-A/D tests. Individuals with Grade 4 hip OA statistically have a significantly longer performance time compared to those with Grade 3 and Grade 2 (p<0.005). This was expected, given that with the progression of the disease, painful symptoms may occur more frequently, and the stiffness and decreased range of hip motion result in walking, bending, or stair negotiation difficulties [19,24].

It was observed that all study groups took longer to ascend stairs than to descend them. This difference may be explained by the fact that during stair ascent, the demands on the joints and muscles are greater to lift the body onto the step [25]. Indeed, biomechanical studies in healthy individuals showed that stair ascent required significantly greater hip flexion angles, maximum hip moments occurred, the stride cycle duration was greater, and the velocity was lower compared to descent, parameters that also apply to hip OA patients [25-27].

According to the literature review, only two studies reported values in hip OA patients [11,12]. In those studies, different stair performance tests than the 9S-A/D were used, and the grade of hip OA was not reported. However, in one study [12], the confirmation of disease severity by the Kellgren-Lawrence classification system was used as an inclusion criterion. Lin et al. [11] used the four-step ascend/descend test, while in the study of Pua et al. [12], the six-step ascend/descend test was implemented. Nevertheless, to compare the results between these studies and ours, each stair test's mean performance time is divided into seconds per step (s/stair). In that case, it follows that the participants of the Lin et al. study [11] ascended stairs at 1.04 s/stair, the same performance time as our Grade 3 participants, but the descend time (1.2 s/stair) was longer by 8.3%, while no ascend/descend time was reported. This difference between the two studies' descent times may be justified by the older age of their participants (69.4 years) compared to our Grade 3, due to the fact that it was reported that stair descent time increases to a greater extent with age than stair ascent time [6]. On the other hand, the Pua et al. [12] study participants had a similar mean age (61 years) to our Grade 2 participants. In this study, only the ascend/descend time was reported (1.03 s/stair), which was 28.2% longer than the performance time of Grade 2 of the present study. The longer performance time may arise because their sample consisted of patients with different severity levels of hip OA (67.1% were classified as Grade 2, 23.9% as Grade 3, and 9% as Grade 4) [12]. It is well known that disease progression increases functional impairments and reduces the ability to perform demanding ADLs such as stair walking [6].

To our knowledge, this is the first study attempting to quantify the diagnostic accuracy of the 9S-ascent, 9S-descent, and 9S-A/D tests. Our ROC analysis corresponding AUCs are above the 0.700 value, showing that 9S-ascent, 9S-descent, and 9S-A/D tests, as markers, have the prognostic ability to excellently distinguish patients according to their grade of hip OA [17]. The ROC analysis results suggest that the 9S-ascent, 9S-descent, and 9S-A/D tests, which are the most demanding tasks among the ADLs [8], could be useful for the early identification of patients with the latter stages of hip OA because the decrease in functionality often appears long before the radiographic classification criteria are reached [4]. Moreover, they provide an easy to be administrated and cost-efficient tool [7], not only for the classification of hip OA grading without the need for radiological findings but also for the frequent re-evaluation of the progression of the disease while the patient follows a more conservative therapeutic protocol, thus minimizing radiation exposure and unnecessary costs.

Regarding the value of the 9S-ascent, 9S-descent, and 9S-A/D tests in the physiotherapy management of hip OA patients, they provide a "ready to go" global evaluation measure of the progression of the disease by monitoring the fluctuation of the test performance times and are able, in real time, to decide if the patient should continue the physiotherapy exercise program or if he should be evaluated for a more interventional approach by his orthopedic surgeon. Specifically, the extracted clinical significance thresholds of this study make the 9S-A/D test (for Grade 2 hip OA patients) or the 9S-descent test (for Grade 3 hip OA patients) an excellent evaluation tool for the effect of the chosen exercise program, the progression of the protocol, and the need, or lack thereof, for further screening or an interventional approach. For the clinical interpretation of those mentioned above, a clinical example follows. Based on a recent systematic review and meta-analysis, exercise therapy in hip OA patients produces actionable results after 5-16 weeks [28]. Assuming a patient with Grade 2 hip OA is following a six-week exercise program. If at three weeks, the 9S-A/D test performance time has decreased by around two seconds [7], compared to the baseline measurement, then the exercise program is adequate, and the patient has benefited; if no difference arises, then the exercise program is inadequate and should be modified accordingly. However, if at six weeks, the 9S-A/D test performance time is almost equal to or increased compared to the baseline measurement, then the patient must be re-evaluated for a more interventional approach by his orthopedic surgeon.

The present study has both strengths and limitations. The study's participants were an adequately representative sample of hip OA patients, the sample size was prodigious, and the statistical analysis was sufficient; these circumstances added power to our results. On the other hand, there are important limitations that have to be mentioned. One limitation of our study is the unequal group size; more participants suffered from Grade 3 hip OA and thus were allocated to Group 3. In ROC analysis, samples with different sizes are considered a classifier bias [29]. The ROC curve can portray an overly optimistic classifier or risk score performance when applied to imbalanced data and unequal groups (classes) [29]. However, we chose not to make the number of participants in each group equal because it's common in the "real world" to have imbalanced data (samples with different sizes), such as in disease diagnosis [30]. Additionally, using ROC analysis gives equal weight to both majority and minority classes, providing an unbiased view of classifier performance without being affected by calibration since it's a ranking-based measurement [30]. Moreover, the study's sample population consisted of patients with primary and unilateral hip OA, without other lower limb joint pain, and with no neurological condition that may affect lower extremity function. Therefore, it must be underlined that the reported performance time values and the cut-off points cannot be generalized to all hip OA patients. It would be interesting to investigate how well ROC analysis quantifies the ability of 9S-ascent, 9S-descent, and 9S-A/D tests to predict the grade of hip OA in future studies involving patients with musculoskeletal or neurological comorbidities.

## Conclusions

In conclusion, the study revealed that as the severity of hip OA increased, the performance time required to complete the 9S-ascent, 9S-descent, and 9S-A/D tests also increased significantly, suggesting that stair negotiation is influenced by the severity of hip OA. Furthermore, ROC analysis revealed that these PPMs, via their cut-off points, have the prognostic ability to excellently distinguish patients according to their hip OA grade. Both classification of the stair performance time values and cut-off points may contribute to monitoring the impact of hip OA progression regarding the patient's functional status and the effect of the chosen therapeutic exercise program or offering the possibility of pre- and post-arthroplasty stair performance comparisons. However, further research is required to report and classify stair performance time values in hip OA patients. Additionally, it is essential to conduct more research focusing on the effectiveness of the 9S-ascent, 9S-descent, and 9S-A/D tests in predicting the severity of hip OA.
